# Adherence to osteoporosis regimens among men and analysis of risk factors of poor compliance: a 2-year analytical review

**DOI:** 10.1186/1471-2474-14-276

**Published:** 2013-09-23

**Authors:** Chun-Kai Chiu, Ming-Chun Kuo, Shan-Fu Yu, Ben Yu-Jih Su, Tien-Tsai Cheng

**Affiliations:** 1 Division of Rheumatology, Allergy and Immunology, E-DA Hospital, I-Shou University, 1 ,Yi-Da Road, Jiau-shu Tsuen, Yan-chau Shiang, Kaohsiung, Taiwan; 2 Division of Endocrinology & Metabolism, Department of Internal Medicine, Kaohsiung Chang Gung Memorial Hospital and Chang Gung University College of Medicine, Kaohsiung, Taiwan; 3 Division of Rheumatology, Allergy and Immunology, Department of Internal Medicine, Kaohsiung Chang Gung Memorial Hospital and Chang Gung University College of Medicine, No 123, Ta-Pei Road, Niaosung, Kaohsiung 833, Taiwan

**Keywords:** Adherence, Osteoporosis regimens, Compliance, Persistence

## Abstract

**Background:**

To investigate adherence and patient-specific factors associated with poor compliance with osteoporosis regimens among men.

**Methods:**

In this retrospective chart review study, we collected data on male patients with osteoporosis treated in accordance with therapeutic recommendations. Adherence was determined by the compliance and persistence of those patients who had been dispensed an osteoporosis regimen after an index prescription. All osteoporosis regimens were considered equivalent for the purpose of investigating adherence.

**Results:**

The prescriptions of 333 males met the inclusion criteria for data collection. The mean age was 68.6 ± 10.4 years. The median medication possession ratio (MPR, %) at years 1 and 2 was 90.1% (interquartile range (IQR) 19–100) and 53.7% (IQR 10.4-100), respectively; 52.3% of male patients at year 1 and 37.5% at year 2 had good compliance (defined as a MPR≧80%). The 1- and 2-year persistence rates were 45.9% and 30.0%, respectively. Patient-specific factors associated with poor compliance (MPR < 80%) during year 1 were first prescriptions given by orthopedists (odds ratio (OR) = 2.67; 95% confidence interval (CI) = 1.58-4.53; adjusted OR = 2.30, 95% CI = 1.26-4.22, p = 0.007). Male patients with rheumatoid arthritis (RA) (OR = 0.22, 95% CI = 0.06-0.78, adjusted OR = 0.19, 95% CI = 0.04-0.81, p = 0.025) and baseline bone mineral density (BMD) measurements (OR = 0.52, 95% CI = 0.32-0.85; adjusted OR = 0.51; 95% CI = 0.28-0.93, p = 0.029) were less likely to have poor compliance.

**Conclusions:**

Adherence to osteoporosis regimens in males was suboptimal in our study. Poor compliance was more likely in prescription of the first anti-osteoporotic regimen by an orthopedist. Men with RA and BMD measurements before therapy had a lower risk of non-adherence. Healthcare professionals need to target patients with specific factors to improve adherence to osteoporotic regimens.

## Background

The prevalence of osteoporosis and osteoporotic fractures has increased worldwide during the past few decades [1]. Osteoporosis and related fractures are a common cause of morbidity and mortality in the elderly globally [2-4]. There are significant differences in skeletal size and structure between men and women that account for differences in fracture incidence, location and outcomes [5]. While women sought medical attention for the prevention of bone loss, men were referred because of the presence of signs and symptoms indicating a more severe disease [6]. In Taiwan, the prevalence of osteoporosis between 1999 and 2001 among subjects aged 50 years or older was 1.63% for men and 11.35% for women [7]. Although the prevalence of osteoporosis is lower in men than in postmenopausal women globally, the mortality and morbidity of osteoporosis among men are higher than among women [5,8-10].

A variety of regimens including bisphosphonates, estrogen, selective estrogen receptor modulators, calcitonin, strontium ranelate, and teriparatide are currently available in Taiwan for the prevention and treatment of osteoporosis, and have demonstrated anti-fracture efficacy in postmenopausal women and in men [11,12]. The efficacy of osteoporosis medications depends on adherence, which consists of both compliance and persistence. Despite the availability of several treatment options for osteoporosis, adherence to anti-osteoporotic therapy in real-world practice is suboptimal and has generally been lower than in clinical trials. In a meta-analysis of 24 studies, 40-70% of osteoporotic patients were reported to be non-adherent [13]. Although male subjects at an advanced age often have osteoporosis and osteoporotic fractures, little is known about their exact adherence rates because researchers rarely include men in osteoporosis adherence studies, [14,15] or seldom report adherence rates separately or exclusively for men. Non-adherence to osteoporosis regimens is clearly associated with poorer outcomes. Therefore, it is essential to improve drug adherence for both postmenopausal women and osteoporotic men, so as to enhance therapeutic outcomes.

Claims or pharmacy databases are usually used to measure adherence and to determine factors associated with non-adherence to osteoporosis medications. Non-adherence in women has been previously reported to be related to the drugs, the patients, and the doctors [16-18]. Few studies in the literature have investigated the factors affecting adherence among males with osteoporosis [14,15]. Therefore, further identification of risk factors for non-adherence among males with osteoporosis may help improve adherence and ultimately reduce fracture risk. In addition, many studies have demonstrated that realizing the anti-fracture efficacy of osteoporosis medications may require several years of therapy [19,20], but most reports have investigated adherence to anti-osteoporotic therapy within one year only [13,21,22].

In this retrospective chart review study of male patients with osteoporosis treated at a single medical center in southern Taiwan, we aimed to investigate 1- and 2-year adherence (both compliance and persistence) to osteoporosis regimens (including bisphosphonates and calcitonin), and also explore the risk factors associated with poor adherence.

## Methods

### Participants

This was a retrospective medical chart review study. The charts of all consecutive male subjects (aged >18 years) who had been diagnosed with osteoporosis and had been dispensed osteoporosis regimens between 1 January 2001 and 31 July 2007 in Chang-Gung Memorial Hospital, Kaohsiung (CGMHK) Medical Center were retrieved. Our hospital is a tertiary care referral center located in Kaohsiung County in southern Taiwan, serving a population of about 2,000,000. The study was conducted according to the protocol approved by the Ethics Committee of Chang Gung Memorial Hospital (IRB No: 97-0165C), and a waiver was granted for obtaining informed consent.

We searched the computerized databases in CGMHK for the following diagnostic codes (International Classification of Diseases, Ninth Edition, Clinical Modification, ICD-9-CM): 733.0 (osteoporosis), 733.01 (senile osteoporosis), 733.02 (idiopathic osteoporosis), 733.03 (disuse osteoporosis), 733.00 (osteoporosis, unspecified), 733.09 (osteoporosis, others), 781.91 (loss of height), V17.81 (family history of osteoporosis), V82.81 (specific screening for osteoporosis), 8054 (spine fracture), and 82,100 (hip fracture). Target osteoporosis regimens included Miacalcic® nasal spray (code: PMF022E, calcitonin 200 iu, Novartis), Fosamax® (10) (code: PMF050M, alendronate, 10 mg tab, Merck Sharp & Dohme), Fosamax® (70) (code: PMF052M, alendronate, 70 mg tab, Merck Sharp & Dohme), and Forteo® (code: PMF042P, teriparatide, 20 mcg/shot, Eli Lilly and Company). Risedronate was not available in Taiwan and ibandronate, zoledronic acid, and strontium ranelate were available only after 2007 in Taiwan. Therefore, the anti-osteoporotic agents targeted in this study did not include these regimens.

### Inclusion and exclusion criteria

Medical records of male subjects who had been dispensed the above regimens under National Osteoporosis Foundation (NOF) treatment recommendations (2008) [23] or the American College of Rheumatology (ACR) guidelines for glucocorticoid-induced osteoporosis (GIOP) (2001) during the study period were reviewed [24]. Patients that did not fulfill the above inclusion criterion, whose chart had been destroyed, who received medications for conditions other than osteoporosis, or those that expired during the follow-up period were excluded.

### Data collection

The demographic and clinical characteristics of the study subjects, including age, gender, selected comorbid conditions, baseline dual energy X-ray absorptiometry (DXA), evidence of prior osteoporotic fracture, sites of osteoporotic fracture, history of vertebroplasty, first prescriber of osteoporosis regimen, and consumption of alcohol/smoking, were examined. Age was assessed at the date of therapy initiation. Comorbidities documentation (including hypertension, cardiac diseases, chronic respiratory diseases, endocrine diseases, chronic kidney disease, hepatobiliary diseases, malignant neoplasms, neuropsychiatric disorders, and rheumatoid arthritis [RA]) and evidence of prior osteoporotic fracture (vertebrae, hip, and other sites of osteoporotic fracture) were ascertained on the basis of the opinion of relevant specialists.

### Calculation of adherence

Adherence, as defined by Cramer *et al.*[25] was evaluated using the parameters of compliance and persistence. Compliance was estimated by the medication possession ratio (MPR) and persistence by the time from treatment initiation to discontinuation with no medication refill gap for a period of 30 days or more during the period of interest. Study patients were rated as having good compliance if the annual MPR ≥80%. MPR was defined as the ratio of actually available doses against the expected doses that the patient should possess over a fixed period of time. The 1-year and 2-year MPR were defined as being the percentage of days with an available drug supply within a predefined interval of 365 days (year 1) or 730 days (year 2), respectively. Persistence rate (PR) was defined as the percentage of patients that were still on medication at a given time with no refill gap in medication-taking for a period of 30 days or more. The risk factors of poor compliance (MPR <80%) were evaluated at the end of year 1. In the sub-group analysis, we excluded those subjects with MPR <80% at the end of year 1 and the MPR of the remaining subjects (MPR ≥80% at year 1) were reevaluated at the end of year 2. The corresponding risk factors were analyzed using the logistic regression model accordingly.

### Statistical methods

Patient characteristics were reported as simple descriptive statistics (i.e., mean ± standard deviation [SD]). The median (25% to 75% inter-quartile range, IQR) was used to summarize data for continuous variables and percentages with a non-normal distribution. In univariate analysis, categorical variables were compared using the chi-square test or Fisher's exact test. Continuous variables were compared using the t-test. The cumulative distribution curves of persistence were generated using the Kaplan-Meier method. Multivariate analysis was performed by estimating the odds ratio (OR) and its 95% confidence interval (CI) using a logistic regression model. All variables of baseline characteristics with a p < 0.05 in univariate analysis were considered for inclusion in the multivariate model. Statistical significance was defined as a p-value of less than 0.05. All analyses were performed using the SPSS program, version 15.5 (SPSS, Chicago, IL) for Windows XP Professional.

## Results

### Patient characteristics

A total of 8280 charts with predefined diagnostic codes were retrieved. After excluding medical records that were not traceable, 3589 charts were reviewed. In all, 333 male subjects met the inclusion criteria for data collection. The mean age of the subjects was 68.6 ± 10.4 years. Of the 333 subjects, 282 (84.7%) fulfilled the NOF recommendation and the remaining (15.3%) fulfilled the ACR guideline. Two thirds of the patients in our cohort had a comorbidity of interest. The most prevalent comorbidity among all patients was hypertension. Most of the patients (90.4%) had a baseline osteoporotic fracture. Vertebral fracture was the most common (79.6%) in our cohort, followed by hip fracture (14.4%). DXA measurement at baseline was available in 28.2% of patients. Rheumatologists and orthopaedists were the most common (72.3 or 72.4%) first prescribers of osteoporosis regimens. Baseline patient characteristics in this study are shown in Table 1.

**Table 1:** Characteristics of the study subjects

**Characteristic**	**Total cohort**	Subgroup*
Number of subjects (male)	333	174
Age (y), mean ± SD	68.6 ± 10.4	67.1 ± 10.9
Comorbidity, n (%)	229 (68.8)	116 (66.7)
Comorbid diseases, n (%)		
1. Hypertension	110 (33.0)	61 (35.1)
2. Neuropsychiatric	58 (17.4)	28 (16.1)
3. Cardiac	52 (15.6)	20 (11.5)
4. Endocrine/metabolic	51 (15.3)	22 (12.6)
5. Respiratory	35 (10.5)	16 (9.2)
6. Malignancy	23 (6.9)	12 (6.9)
7. Chronic kidney disease	22 (6.6)	10 (5.7)
8. Hepatobiliary	21 (6.3)	15 (8.6)
9. Rheumatoid arthritis	17 (5.1)	14 (8.0)
Fracture history, n (%)	301 (90.4)	151 (86.8)
Fracture sites, n (%)		
Spine	265 (79.6)	134 (77.0)
Hip	48 (14.4)	21 (12.1)
Others^@^	21 (6.3)	8 (4.6)
Baseline DXA, n (%)	94 (28.2)	60 (34.5)
BMD T-score (SD), mean ± SEM		
Spine	−2.0 ± 0.2	−1.9 ± 0.2
Femoral neck	−2.1 ± 0.1	−1.9 ± 0.1
Total hip	−1.7 ± 0.1	−1.6 ± 0.2
First prescriber, n (%)		
Rheumatology	140 (42.0)	85 (48.9)
Orthopedics	101 (30.3)	37 (21.3)
Others^#^	92 (27.6)	52 (29.9)
Enrolment criteria		
NOF recommendation, n (%)	282 (84.7)	144 (82.8)
ACR guideline, n (%)	51 (15.3)	30 (17.2)
Vertebroplasty, n (%)	11 (3.3)	6 (3.4)
Tobacco use	1 (0.3)	0 (0)
Alcohol use	2 (0.6)	1 (0.6)

*Subjects with MPR ≥ 80% at year 1; NOF (2008): Clinician's Guide to Prevention and Treatment of Osteoporosis by the National Osteoporosis Foundation (2008); ACR (2001): Recommendations for the treatment and prevention of glucocorticoid-induced osteoporosis by the American College of Rheumatology (2001); DXA: Dual energy X-ray absorptiometry; BMD: bone mineral density; SD: standard deviation; SEM: standard error of the mean; Serum creatinine >1.4 mg/dl; ^@^: including rib, wrist, upper arm and pelvis; ^#^: Physician of Family Medicine, Metabolism, Rehabilitation.

### Drug adherence and patient-specific factors associated with poor compliance

In terms of compliance, the median MPR at year 1 and year 2 was 90.1% (IQR 19–100) and 53.7% (IQR 10.4-100), respectively. More than half (52.3%) of the males had good compliance (MPR ≥ 80%) at year 1, but only 37.5% through year 2. In addition, the PR of all 333 men at year 1 and year 2 was 45.9% and 30.0%, respectively**.** One-year compliance was lower with start prescriptions by orthopedists (OR = 2.673, p = 0.000; CI, 1.58-4.53; adjusted OR = 2.302, CI, 1.26-4.22, p = 0.007, compared to start prescriptions by rheumatologists). Male patients with RA (OR = 0.22, 95% CI = 0.06-0.78, adjusted OR = 0.19, 95% CI = 0.04-0.81, p = 0.025) and baseline bone mineral density (BMD) measurements (OR = 0.52, 95% CI = 0.32-0.85; adjusted OR = 0.51; 95% CI = 0.28-0.93, p = 0.029) were less likely to have poor compliance (Table 2). There was no association between the other baseline characteristics and poor compliance at year 1.

**Table 2:** Factors associated with poor-compliance in subjects of total cohort and subgroup

	**Total subjects at year 1 (n = 333)**					**Subgroup subjects at year 2 (n = 174)**^**a**^				
**Risk factors**	**n (%)**	**Univariate analysis**		**Multivariate analysis**		**n (%)**	**Univariate analysis**		**Multivariate analysis**	
		**OR 95% CI**	**p-value**	**OR 95% CI**	**p-value**		**OR 95% CI**	**p-value**	**OR 95% CI**	**p-value**
Age		1.03 (1.01-1.05)	0.007	1.02 (0.99-1.05)	0.132		0.97 (0.94-0.99)	0.045	0.97 (0.94-1.01)	0.100
First prescribed by										
Rheumatologist	55 (34.6)	1.0 (referent)		1.0 (referent)		24 (48.0)	1.0 (referent)		1.0 (referent)	
Orthopedist	64 (40.3)	2.67 (1.58-4.53)	0.000	2.30 (1.26-4.22)	0.007	13 (26.0)	1.38 (0.60-3.14)	0.447	1.52 (0.58-3.96)	0.394
Others	40 (25.2)	1.19 (0.70-2.03)	0.525	1.12 (0.62-2.05)	0.709	13 (26.0)	0.85 (0.39-1.86)	0.679	0.86 (0.35-2.14)	0.746
DXA	13 (8.2)	0.52 (0.32-0.85)	0.008	0.51 (0.28-0.93)	0.029	17 (34.0)	0.97 (0.49-1.94)	0.932	0.94 (0.39-2.26)	0.895
Vertebroplasty	5 (3.1)	0.91 (0.27-3.04)	0.877	0.59 (0.15-2.30)	0.450	1 (2.0)	0.49 (0.06-4.27)	0.515	0.71 (0.07-7.08)	0.769
Fracture site										
Spine fracture	131 (82.4)	1.40 (0.81-2.40)	0.225	1.54 (0.45-5.29)	0.489	36 (72.0)	0.68 (0.32-1.45)	0.320	0.37 (0.05-2.95)	0.345
Hip fracture	27 (17.0)	1.49 (0.81-2.76)	0.204	1.21 (0.41-3.54)	0.731	7 (14.0)	1.28 (0.48-3.36)	0.620	0.47 (0.06-3.50)	0.457
Others fracture	13 (8.2)	1.85 (0.75-4.58)	0.185	1.88 (0.69-5.10)	0.216	3 (6.0)	1.52 (0.35-6.61)	0.577	1.16 (0.22-6.23)	0.863
Tobacco use^b^	1 (0.6)	--	0.477	0.00 (0.00)	1.000	0 (0)	--	--	--	--
Alcohol use	1 (0.6)	1.10 (0.07-17.65)	0.949	0.00 (0.00)	1.000	1 (2.0)	4.09 (0.00)	1.000	1.37 (0.00)	1.000
Malignancy	11 (6.9)	1.00 (0.43-2.34)	0.994	0.98 (0.39-2.45)	0.960	3 (6.0)	0.82 (0.21-3.15)	0.767	0.78 (0.19-3.27)	0.734
Cardiac disease	32 (20.1)	1.94 (1.06-3.56)	0.032	1.87 (0.93-3.75)	0.079	7 (14.0)	1.39 (0.52-3.72)	0.512	1.79 (0.58-5.49)	0.311
Respiratory disease	19 (11.9)	1.34 (0.66-2.71)	0.413	1.22 (0.57-2.63)	0.610	4 (8.0)	0.81 (0.25-2.65)	0.729	0.72 (0.20-2.56)	0.611
Neuropsychiatric disease	30 (18.9)	1.21 (0.69-2.14)	0.505	0.89 (0.47-1.70)	0.732	6 (12.0)	0.63 (0.24-1.67)	0.354	0.68 (0.23-2.02)	0.485
Chronic kidney disease	12 (7.5)	1.34 (0.56-3.19)	0.509	1.35 (0.51-3.58)	0.548	2 (4.0)	0.60 (0.12-2.95)	0.533	0.66 (0.12-3.62)	0.636
Endocrine/metabolic	29 (18.2)	1.54 (0.85-2.81)	0.157	1.81 (0.90-3.64)	0.095	6 (12.0)	0.92 (0.34-2.51)	0.871	0.82 (0.26-2.60)	0.734
Hypertension	49 (30.8)	0.83 (0.52-1.31)	0.411	0.62 (0.37-1.06)	0.083	21 (42.0)	1.52 (0.77-2.99)	0.224	1.62 (0.77-3.43)	0.207
Rheumatoid arthritis	3 (1.9)	0.22 (0.06-0.78)	0.011	0.19 (0.04-0.81)	0.025	4 (8.0)	0.99 (0.30-3.32)	0.989	0.81 (0.17-3.89)	0.790
Hepatobiliary disease	6 (3.8)	0.42 (0.16-1.10)	0.075	0.44 (0.16-1.25)	0.124	2 (4.0)	0.36 (0.08-1.64)	0.185	0.45 (0.09-2.33)	0.340
Steroid use	21 (13.2)	0.73 (0.40-1.34)	0.307	1.48 (0.68-3.22)	0.320	10 (20.0)	1.30 (0.56-3.02)	0.541	1.02 (0.31-3.29)	0.980

*OR* odds ratio, *CI* confidence interval, Serum creatinine >1.4 mg/dl; ^a^Subjects with MPR ≥ 80% at year 1; ^b^No one smoked, so the factor cannot be estimated; All comparisons denote present *vs*. absent.

In addition, we also analyzed the subgroup with a MPR ≥ 80% at year 1 to recognize the predictors of non-adherence at year 2. Baseline patient characteristics in the subgroup (n = 174) are shown in Table 1. No identified factor was associated with poor compliance during year 2 in this subgroup of subjects (Table 2).

## Discussion

Osteoporosis in men has been documented as a serious health issue recently, as osteoporosis can cause higher morbidity and mortality in men than in women, though it affects males less frequently [5,10]. Indeed, whereas numerous data related to the adherence of female osteoporosis patients are available, data are scarce for men. To our knowledge, the present study is one of few that have investigated the factors affecting medication compliance among males with osteoporosis. The findings were in accord with the results of our total cohort [26], of which more than 90% of subjects were female: adherence among male subjects was suboptimal. In contrast with previous investigations of female osteoporosis, our survey revealed the major risk factors of poor compliance at year 1 were index prescription by orthopedists and not having undergone baseline BMD measurement before pharmacologic treatment for osteoporosis was initiated. However, male subjects with RA were more likely to have good compliance.

Compliance with medications used to treat or prevent osteoporosis ranged from 34% to 94.6% at 1 year [27-31] and 27% to 43% at 2 years [27,32]. However, the results were subject to various definitions of compliance, and female gender, bisphosphonate users and different study populations were mainly focused on in each investigation [28,30]. Our data examined adherence not only among male users of oral (daily and weekly) bisphosphonates, but also among male users of calcitonin. Hansen et al. [14] reported that adherence to alendronate in male veterans during the first 12 and 24 months of therapy was 59% and 54%, respectively. A small series study by Cevikoi A [15] demonstrated that full compliance rates for the 1- and 3-year periods were 49.4% and 17.9%, respectively. The results of our investigation of good compliance at the first year were similar to those of most studies on postmenopausal women [14,32,33]. However, at year 2, good compliance in our cohort was 37.5%, which was lower than that in Hansen’s study [14]. A recent study on compliance among male subjects reported that the 1-year good compliance (MPR ≥ 80%) and persistence rates were 91% and 39.7%, respectively [21]. They attributed the relatively high 1-year good compliance rate to (1) including both new and existing patients with at least 3 prescriptions; (2) the specific ethnic group (a Dutch population); and (3) reimbursement of all osteoporosis medications [21]. That was in contrast to our study population, which had fewer than 3 prescriptions and limited reimbursement for fracture subjects.

Previous investigations that were mainly focused on bisphosphonate users demonstrated 1-year PRs ranging from 24% to 74.8% [15,21,34-36]. In our study, the persistence curve revealed an obvious drop (38.3%) in the first year, but a less prominent (19.5%) drop in the second year among those subjects persistent with their medication at the end of the first year. This was similar to the observation in several studies on adherence to anti-osteoporosis agents among postmenopausal women [37-39]. The previous study had demonstrated that increasing age, pain, no use of diagnostic tests, and male showed a positive effect on the probability of quitting the anti-osteoporotic medication [40]. It also suggests that improving adherence in the first year is essential to achieving a better therapeutic response for either gender. Therefore, the factors related to inadequate compliance for each year were investigated in the current study.

Possible factors affecting medication adherence have been reviewed in the literature and include side effects, smoking behavior, surveying for bone density during therapy, educational status, social support, marital status, and income [14,15,41]. However, these factors were not analyzed annually, as done in our investigation. Data on several characteristics of male subjects, e.g., jobs, lifestyle, and educational levels, were not collected, which potentially may be related to compliance with therapy in the current study. Hence, our finding suggests that certain unidentified factors pertaining to male subjects may be related to poor compliance, as demonstrated by previous studies.

In our study, patients of orthopedists were more likely to discontinue osteoporosis medications at year 1 than those seeing rheumatologists. This is in agreement with a previous study showing that patients of orthopedists were more likely to discontinue their medications than the patients of rheumatologists and general practitioners, probably due to a lack of adequate doctor motivation [42]. Physician specialty was significantly associated with adherence to treatment guidelines [43]. Ideguchi *et al.* found that patients of gynecologists or rheumatologists were more likely to continue bisphosphonates than patients of orthopedists or other specialists [44]. BMD measurement was highest among patients treated with glucocorticoids prescribed by rheumatologists [45]; therefore, rheumatologists often had more opportunities to prescribe anti-osteoporotic medications to prevent or treat GIOP than other specialties. We speculated from our study results that having follow-up for each long-term prescription by a physician specializing in osteoporosis (especially an internist, for example, a rheumatologist) with feedback to the patients may improve the patient-physician relationship and adherence.

A lack of DXA at baseline (before therapy) was another factor of 1-year poor compliance in our study. Similar observations were also reported in Pickney’s [46] and Solomon’s studies [29]. In contrast, Hansen et al. showed that non-adherence to oral anti-osteoporosis medications among men was associated with a lack of measurements of bone mass during alendronate therapy, but not before therapy [14]. It has been demonstrated that a correct understanding of BMD results would lead to higher treatment rates and better adherence to treatment [46]. Adherers who have undergone bone mass measurements at baseline might be more aware of their disease course and of the risks of subsequent or recurrent fractures than non-adherers.

Richards *et al.* observed that non-adherence to bisphosphonates was common in a cohort of RA patients who were veterans in the United States [47]. In contrast, RA patients in our study were more likely to continue osteoporosis medications. We would expect good compliance with osteoporosis medications in patients with RA because of the awareness of physicians and patients about the negative effects of glucocorticoids on bone. Most patients with RA may need to receive steroid treatment in clinical practice, which puts them at high risk for GIOP. Rossini *et al.* demonstrated positive associations between glucocorticoid and anti-inflammatory treatment and compliance with osteoporosis medications [42]. Although steroid users did not have a higher compliance rate in the current study, other determining factors associated with adherence among osteoporotic RA male patients could be further explored in the future.

There are several limitations in our study. (1) It is unknown if patients actually took the dispensed drug. We assumed that patients who obtain prescription refills do take their medications based on chart review. In addition, in Taiwan, patients can obtain 2 to 3 prescriptions each visit for a stable disease. We were unable to ascertain whether patients obtained refills at outside pharmacies. As a result, adherence may be overestimated. (2) We determined adherence based on the number of refills. Therefore, whether the drug was delivered correctly at the correct time of day or in appropriate doses is unknown. (3) Due to the retrospective study design, some information obtained from chart reviews may be not complete and the data collection may be subjective. (4) We did not collect data on the side effects of anti-osteoporotic regimens from the chart reviews, which may actually have influenced adherence. (5) Our sample size was relatively small. Therefore, we may not have determined all factors associated with non-adherence among male patients. (6) Finally, patient characteristics linked to adherence from databases at a single medical center probably were not universally applicable to men treated by community-based practitioners.

However, our study has some advantages. (1) We reviewed medical charts to determine patient variables associated with adherence, information that is often not acquired in claims databases. (2) Most studies reported on the adherence mainly of postmenopausal women; we targeted men who are not normally enrolled or included in osteoporosis adherence studies exclusively. (3) We analyzed adherence over a period of 2 years and identified patient-specific factors of poor adherence on a year-by-year basis. This information can serve as a reminder to prescribers year by year to improve adherence.

## Conclusions

Our study indicates that men with osteoporosis are not optimally treated in daily clinical practice in southern Taiwan. Starting prescriptions by orthopedists significantly contributed to non-compliance, and men with RA and baseline bone mass measurements were less likely to have poor compliance. Identifying risk factors associated with non-adherence may help healthcare professionals improve adherence.

## Abbreviations

(MPR): Median medication possession ratio; (IQR): Interquartile range; (OR): Odds ratio; (CI): Confidence interval; (BMD): Bone mineral density; (RA): Rheumatoid arthritis; (CGMHK): Chang-Gung Memorial Hospital, Kaohsiung; (ICD-9-CM): International Classification of Diseases, Ninth Edition, Clinical Modification; (NOF): National Osteoporosis Foundation; (ACR): American College of Rheumatology; (GIOP): Glucocorticoid-induced osteoporosis; (DXA): Dual energy X-ray absorptiometry; (PR): Persistence rate; (SD): Standard deviation.

## Competing interests

The authors have indicated that they have no conflict of interest regarding the content of this report.

## Authors’ contributions

All authors made substantive intellectual contributions to this study to qualify as authors. CC and MK contributed to study design, acquisition of data, analysis of data, and interpretation of results. SY and TC contributed to study coordination. BYS contributed to statistical analysis. CC, MK, SY and TC contributed to manuscript preparation. All authors read and approved the final manuscript.

## Pre-publication history

The pre-publication history for this paper can be accessed here:

http://www.biomedcentral.com/1471-2474/14/276/prepub
